# Regular exercise combined with ferulic acid exhibits antiobesity effect and regulates metabolic profiles in high-fat diet-induced mice

**DOI:** 10.3389/fnut.2022.957321

**Published:** 2022-07-27

**Authors:** Ou Wang, Nanhai Zhang, Chao Han, Jian Huang

**Affiliations:** ^1^National Institute for Nutrition and Health, Chinese Center for Disease Control and Prevention, Beijing, China; ^2^Key Laboratory of Trace Element Nutrition of National Health Commission of People's Republic of China, Beijing, China; ^3^Beijing Key Laboratory of Functional Food From Plant Resources, College of Food Science and Nutritional Engineering, China Agricultural University, Beijing, China

**Keywords:** ferulic acid, exercise, obesity, metabolic profiles, lipid metabolism, oxidative stress

## Abstract

Exercise (Ex) has been recognized as an effective way of obesity prevention, but it shows a dual effect on the body's antioxidant system. Ferulic acid (FA) is a kind of phenolic acid with well-known antioxidant capacity and numerous health benefits. Therefore, the aim of the study was to compare the antiobesity effect of Ex, FA, and Ex combined with FA (Ex-FA) *in vivo* and to illustrate the potential mechanisms. Mice were fed a high-fat diet (HFD) with or without administration of Ex, FA, and Ex-FA for 13 weeks. The body weight, antioxidant ability, Ex performance, and lipid profiles in the serum, liver, and skeletal muscle were compared among the groups, and serum metabolomics analysis was conducted. The results showed that Ex, FA, and Ex-FA exhibited a similar effect on body weight management. Ex had a more beneficial function by alleviating HFD-induced dyslipidemia than FA, while FA exerted a more efficient effect in mitigating lipid deposition in the liver and skeletal muscle. Ex-FA showed comprehensive effects in the regulation of the lipid contents in serum, liver, and skeletal muscle, and provoked enhancement effects on antioxidant ability and Ex capacity. Mice administered with Ex, FA, and Ex-FA showed different metabolic profiles, which might be achieved through different metabolic pathways. The findings of this research implied that Ex coupled with FA could become an effective and safe remedy for the management of dietary-induced obesity.

## Introduction

Exercise (Ex) is a highly recommended lifestyle for the prevention or attenuation of obesity (1). It also exerts biological benefits for regulation of glucose and lipid metabolism (2), enhancement of skeletal muscle function (3), and protection of cardiac health (4). With the popularity of Ex, there has been an increasing market demand for sports nutrition food. To date, the dominant ingredient of sports nutrition food is protein, which accounts for more than 80% of the market (5).

Ex with different styles and intensities can affect the body's antioxidant system and people's health to varying degrees (6). The moderate Ex can protect active people from oxidative stress (7), while the long-term chronic training results in the increase of oxidative damage biomarkers (8). Besides, intensive physical activity can lead to an imbalance of endogenous oxidants and antioxidants, thus causing systemic oxidative stress (9), skeletal muscle fatigue, and further oxidative modification of proteins, lipids, and DNA (10). In view of the relationship of Ex and oxidative stress, the application of antioxidants seems to be a necessary therapy in sports nutrition and health (11). It has been proved by a human studies that the oral intake of antioxidants could effectively relieve Ex-induced oxidative stress (12, 13).

Ferulic acid (FA) is a kind of phenolic acids that is widely presented in natural food, like grains, coffee, and berry fruits. Similar to other polyphenol compounds, FA has an effect on scavenging free radicals both *in vitro* and *in vivo* (14, 15). The previous study suggested that FA could alleviate dietary-induced metabolic syndrome (16), improve insulin sensitivity in skeletal muscle and hypothalamus (17), and promote hypertrophic growth of skeletal muscle (18). Based on these researches, it can be speculated that FA might not only show similar effects as Ex in attenuating metabolic disorders and inhibiting obesity, but also exhibit antioxidant properties in mitigating the Ex-induced oxidative stress. In addition, whether FA can strengthen the antiobesity effect of Ex is poorly understood.

The aim of this research was to compare the effects of Ex, FA, and Ex combined with FA on the high-fat diet (HFD)-induced obesity in mice. The fat distribution, Ex performance, and oxidative stress status among the groups were compared, and the metabolic profiles were analyzed to illustrate the potential mechanisms. The results of this research might provide a scientific basis for the utilization of FA in the functional sports nutrition food industry.

## Materials and methods

### Materials and chemical reagents

FA (purity 98%) was purchased from Nanjing Jingzhu Biotechnology Co., Ltd. (Nanjing, China). The normal fat diet (ND, D12450B, 3.85 kCal/g, 4.3% fat, 67.3% carbohydrate, and 19.2% protein, w/w) and HFD (D12492, 5.24 kCal/g, 60% fat, 20% carbohydrate, and 20% protein) were provided by the Beijing Keaoxieli Feed Co., Ltd. (Beijing, China). The test kits for malondialdehyde (MDA) and free fatty acid (FFA) were purchased from the Nanjing Jiancheng Bioengineering Institute (Nanjing, China). Triglycerides (TG), total cholesterol (TC), high-density lipoprotein–cholesterol (HDL-C), and low-density lipoprotein–cholesterol (LDL-C) assay kits were purchased from the Wako Pure Chemical Industries, Ltd. (Osaka, Japan). Reagents for reactive oxygen species (ROS) staining were obtained from the Wuhan Servicebio Technology Co., Ltd. (Wuhan, China). All the other chemical reagents were of analytical or chromatographic grade and purchased from the Sinopharm Group Co., Ltd. (Beijing, China).

### Animal and treatments

Male C57BL/6J mice (18–20 g, SPF grade) were obtained from the Beijing Huafukang Bioscience Co., Ltd. (Beijing, China) (Certificate SCXK 2019-0008). Mice were housed in an SPF environment with a controlled temperature of 25 ± 1°C and relative humidity of 50 ± 5%. The light and dark cycle was 12 h (8:00 a.m. to 8:00 p.m.). After 1 week of acclimatization, mice were randomly divided into five groups (*n* = 10) and received different treatments for 13 weeks. Mice in the control group (ND) and model group (HFD) were fed ND and HFD, respectively. Mice in the Ex group (Ex) were fed HFD and received regular Ex. Mice in the FA group (FA) were fed HFD and given FA by an oral route. Mice in the Ex combined with FA group (Ex-FA) were fed HFD and received both of regular Ex and FA administration. During the entire experiment, mice were given free access to clean water and food. The food intake was calculated every 2 weeks.

For the ND, HFD, and Ex groups, mice were administrated 0.5% sodium carboxymethyl cellulose solution by oral gavage. For the FA and Ex-FA groups, mice were supplied orally with 136 mg/kg body weight of FA (suspended in 0.5% sodium carboxymethyl cellulose solution) 7 days per week. Mice were weighed weekly, and the gastric infusion volume was adjusted with their body weight. For the Ex-FA group, mice were treated with FA after Ex.

For the regular Ex, mice ran on a motor-driven treadmill (ZS-PT-III, Beijing, China) at a speed of 10 m/min for 10 min per day for five successive days for adaptation and then got involved in the regular Ex. From week 1 to week 3, mice were trained at a speed of 10 m/min for 30 min per day. From week 4 to week 6, mice were trained for 40 min per day (10 m/min for 20 min and then uniformly accelerated from 10 m/min to 18 m/min within 20 min). From week 7 to week 13, mice were trained for 60 min per day (10 m/min for 20 min, then uniformly accelerated from 10 m/min to 18 m/min within 20 min, and finally 18 m/min for 20 min). Mice were trained for five successive days per week and rested for the other two days.

All the animal procedures were performed in accordance with the Guidelines for Animal Experiment of China and approved by the Laboratory Animal Welfare and Ethics Committee of the National Institute for Nutrition and Health, Chinese Center for Disease Control and Prevention (IACUC No. 2020-NINH-IACUC-008).

### Magnetic resonance imaging (MRI)

In the middle of the 13th week, proportions of the body fat and skeletal muscle in mice were analyzed by MRI. Briefly, mice were anesthetized by oxygen with 2% isoflurane at a flow rate of 1 L/min, and then scanned using a 7.0 T small animal MRI system (Agilent, Palo Alto, CA, USA). The images were captured with a T1-spin echo multislice sequence as follows: field of view =96.7 × 58.6 mm, no slices =34, slice thickness =0.6 mm (zero slice gap), repetition time =665.99 ms, echo time =13.44 ms, image matrix = 256 × 256, number of averages =6. The images were analyzed by Inveon Research Workplace 4.2 (Siemens, Germany), and percentages of the body fat and hindlimb skeletal muscle in mice were calculated.

### Exercise performance test

On the first day of the 13th week, mice in all the groups ran on a treadmill at a beginning speed of 10 m/min for 5 min, and then at an evenly increasing speed from 10 m/min to 18 m/min within 20 min on a 5° slope. Finally, mice ran at a speed of 18 m/min on a 5° slope until exhausted. The status of exhaustion was defined when mice stopped running, even driven by hand. The distance of mice running at 18 m/min on a 5° slope until exhausted was recorded as the movement distance.

On the third day of the 13th week, the forelimb grip strength of mice was assayed using a grip tester (ZS-ZL, Beijing, China). In short, the forelimbs of mice held the mesh grip board and then dragged the tail with a force parallel to the grip board until the mice fell off, then a force would display on the instrument, and recorded as the grip force. All mice were acclimated before the formal test.

### Organ indexes and biochemical parameters determination

At the end of the 13th week, mice were fasted overnight and sacrificed. Blood samples were collected from the orbital plexus. Serum was isolated from the blood samples by centrifugation at 3,500 *g* for 15 min at 4°C. The levels of TG, TC, HDL-C, and LDL-C in the serum were analyzed on a 7,600 automatic biochemical analyzer (Hitachi, Japan) using corresponding assay kits. After sacrificing, liver, epididymal fat, abdominal fat, and brown fat were removed and weighed immediately to calculate the organ indexes. The gastrocnemius muscles were isolated from the hindlimbs of mice. Liver and muscle tissues were mixed with ice-cold normal saline at a mass-to-volume ratio of 1:9 and homogenized by a T10 homogenizer (IKA, Staufen, Germany). The supernatant was collected after centrifugation at 3,500 *g* for 15 min at 4°C for the detection of TC, TG, FFA, and MDA levels using corresponding kits.

### ROS analysis

The muscle tissues were frozen in liquid nitrogen, sliced, and stained with ROS staining solution and 4,6-diamino-2-phenylindole (DAPI) solution. Images were captured by fluorescent microscopy and analyzed with Image J 1.50i (NIH, USA). Nucleus labeled with DAPI were blue, and ROS-positive areas were red.

### Metabolomics analysis

Serum samples (*n* = 7 for each group) were randomly selected for the untargeted metabolomics analysis. Simply, 100 μL serum was mixed with 400 μL methanol-acetonitrile solution (1:1, v/v) with L-2-chlorophenylalanin as the internal standard. After well mixed, the mixture was ultrasonically treated at 40 kHz for 30 min at 5°C. The mixing solution was placed at 20°C for 30 min to precipitate the proteins in the serum samples. Then the supernatants were collected after centrifugation at 13,000 g for 15 min at 4°C and transferred to sample vials for LC-MS/MS analysis.

The LC-MS/MS analysis was performed on the UHPLC-Q Exactive HF-X system equipped with an ACQUITY UPLC HSS T3 column (100 mm × 2.1 mm i.d., 1.8 μm; Waters, Milford, USA) maintained at 40°C. Gradient elution was executed with 0.1% formic acid in acetonitrile–water solution (5:95) (A) and 0.1% formic acid in a mixture of acetonitrile, isopropanol, and water (47.5:47.5:5) (B). After equilibration, 2 μL of each sample was injected. The gradient of solvent B (v/v) was added as follows: 0–3.5 min, 0–24.5% B (0.4 mL/min); 3.5–5.0 min, 24.5–65% B (0.4 mL/min); 5.0–5.5 min, 65–100% B (0.4 mL/min); 5.5–7.4 min, 100% B (0.4–0.6 mL/min); 7.4–7.6 min, 100–51.5% B (0.6 mL/min); 7.6–7.8 min, 50.5–0% B (0.6–0.5 mL/min); 7.8–9.0 min, 0% B (0.5–0.4 mL/min); 9.0–10.0 min, 0% B (0.4 mL/min). Mass spectra were acquired with a spray voltage of −3.5 kV and 3.5 kV in negative and positive modes, respectively. The flow rates of sheath gas and auxiliary gas were set at 50 and 13 arbitrary units, respectively. The heater temperature and capillary temperature were set at 425 and 325°C, respectively. The analyzer scanned over a mass range of *m*/*z* 70–1,050 for a full scan at a mass resolution of 60,000. The normalized collision energy was rolling as 20-40-60 V.

The raw data of metabolomics were analyzed on the online platform Majorbio Cloud Platform (www.majorbio.com, Shanghai Majorbio Bio-pharm Technology Co., Ltd, China) (19). Briefly, after data preprocessing and annotation, partial least squares-discriminant analysis (PLS-DA) and orthogonal partial least squares discriminant analysis (OPLS-DA) were used to distinguish the overall differences among groups. The differential metabolites were preliminarily identified based on variable importance in the projection (VIP) values (>1.0) and statistical analysis (*P* < 0.05). The final identification and analysis of implicated pathways associated with the metabolites were carried out using databases, including the Human Metabolome Database (HMDB) and the Kyoto Encyclopedia of Genes and Genomes (KEGG).

### Statistical analysis

All the data from animal experiments were shown as mean ± standard deviation (SD) and analyzed by SPSS 22.0 (Inc., Chicago, CA, USA). One-way analysis of variance (ANOVA) followed by Tukey's test was used to evaluate the statistical significance among groups. Differences were considered significant when *P* < 0.05.

## Results

### Effects of Ex, FA, and Ex-FA on body weight and organ indexes in HFD-fed mice

**Figure 1:**
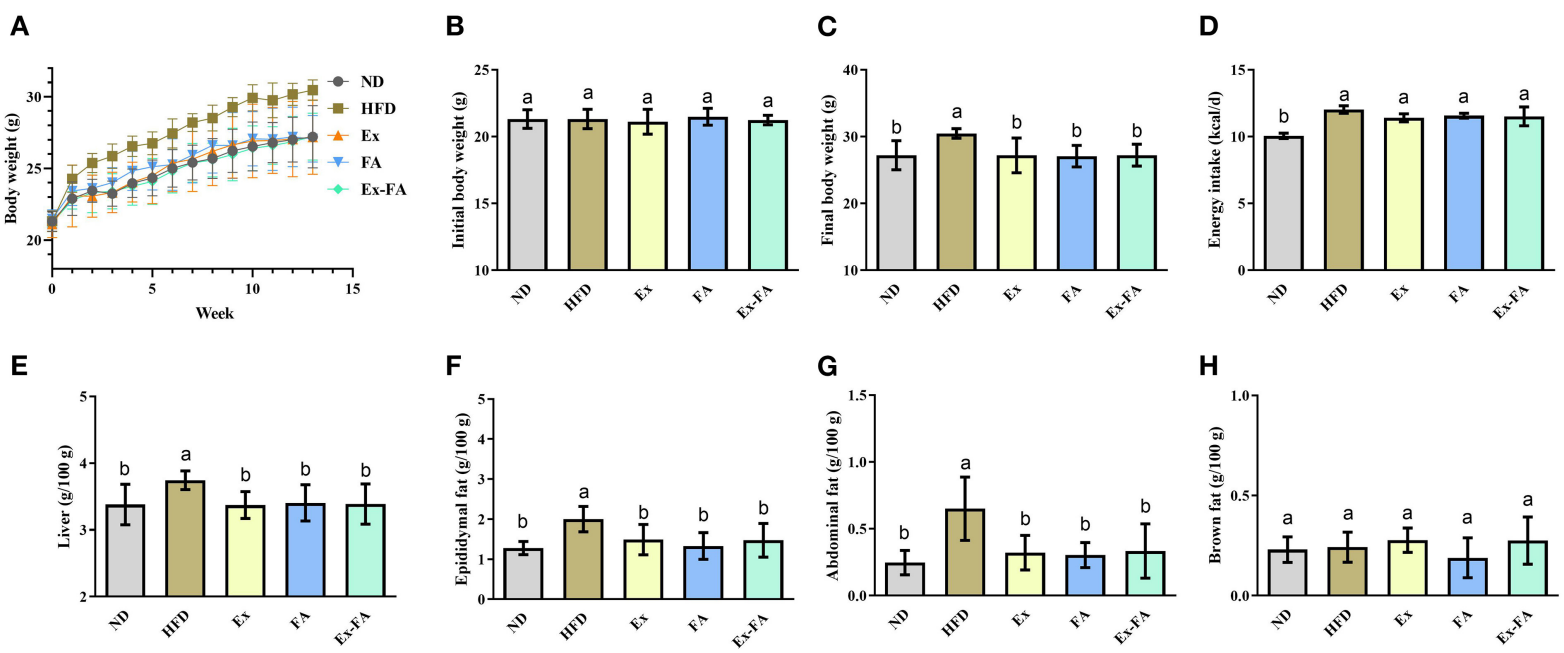
Effects of Ex, FA, and Ex-FA on body weight, energy intake, and organ indexes in HFD-fed mice. **(A)** Body weight change; **(B)** Initial body weight; **(C)** Final body weight; **(D)** Energy intake; **(E)** Liver index; **(F)** Epididymal fat index; **(G)** Abdominal fat index; **(H)** Brown fat index. ND, normal diet group (control); HFD, high-fat diet group (model); Ex, high-fat diet with exercise; FA, high-fat diet with ferulic acid; Ex-FA, high-fat diet with exercise and ferulic acid. Data are expressed as mean ± SD (*n* = 6 for energy intake and *n* = 10 for the others). Means with different letters are significantly different (*P* < 0.05).

**Figure 2:**
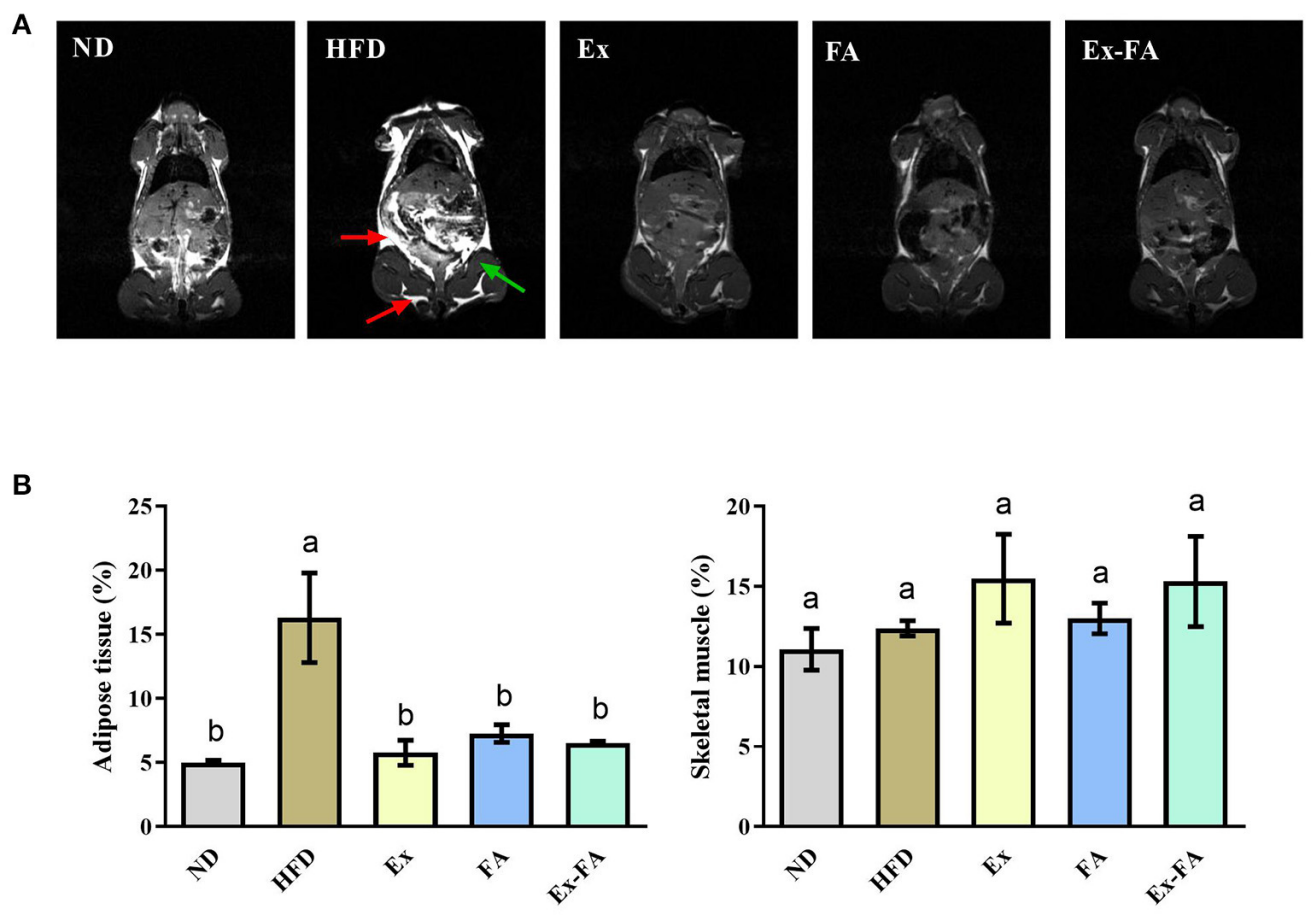
Effects of Ex, FA, and Ex-FA on body composition in HFD-fed mice. **(A)** Magnetic resonance images; **(B)** Proportions of adipose tissue and skeletal muscle. The proportions of adipose tissue and skeletal muscle were calculated from the magnetic resonance images. The red arrow represents adipose tissues, and the green arrow represents the hindlimb skeletal muscle. ND, normal diet group (control); HFD, high-fat diet group (model); Ex, high-fat diet with exercise; FA, high-fat diet with ferulic acid; Ex-FA, high-fat diet with exercise and ferulic acid. Data are expressed as mean ± SD (*n* = 3). Means with different letters are significantly different (*P* < 0.05).

According to the data in Figure 1, mice in the HFD group showed significantly higher final body weight, hepatic index, epididymal fat index, and abdominal fat index than mice in the ND group (*P* < 0.05). After MRI quantification, the proportion of adipose tissues in HFD-fed mice was also significantly higher than that in the ND group (*P* < 0.05; Figure 2). Therefore, HFD administration for 13 weeks could induce obesity in mice.

Compared with the mice in the HFD group, Ex, FA, and Ex-FA treatments markedly decreased the final body weight and indexes of liver and white fat tissues at the end of the 13th week (*P* < 0.05; Figure 1). In the MRI scanning images, the adipose tissue areas in Ex-, FA-, and Ex-FA-treated mice were smaller than that in the HFD group, and the proportion of adipose tissues was markedly reduced compared to that in the HFD group (*P* < 0.05; Figure 2). Besides, the energy intake in the Ex, FA, and Ex-FA groups showed no apparent difference from that in the HFD group (Figure 1). This finding indicated that the food intake and appetite were not affected by the treatments of Ex, FA, and Ex-FA. Moreover, no obvious difference was found in the brown fat index and proportion of the hindlimb skeletal muscle among all the groups (Figures 1, 2).

### Effects of Ex, FA, and Ex-FA on exercise performance in HFD-fed mice

**Figure 3:**
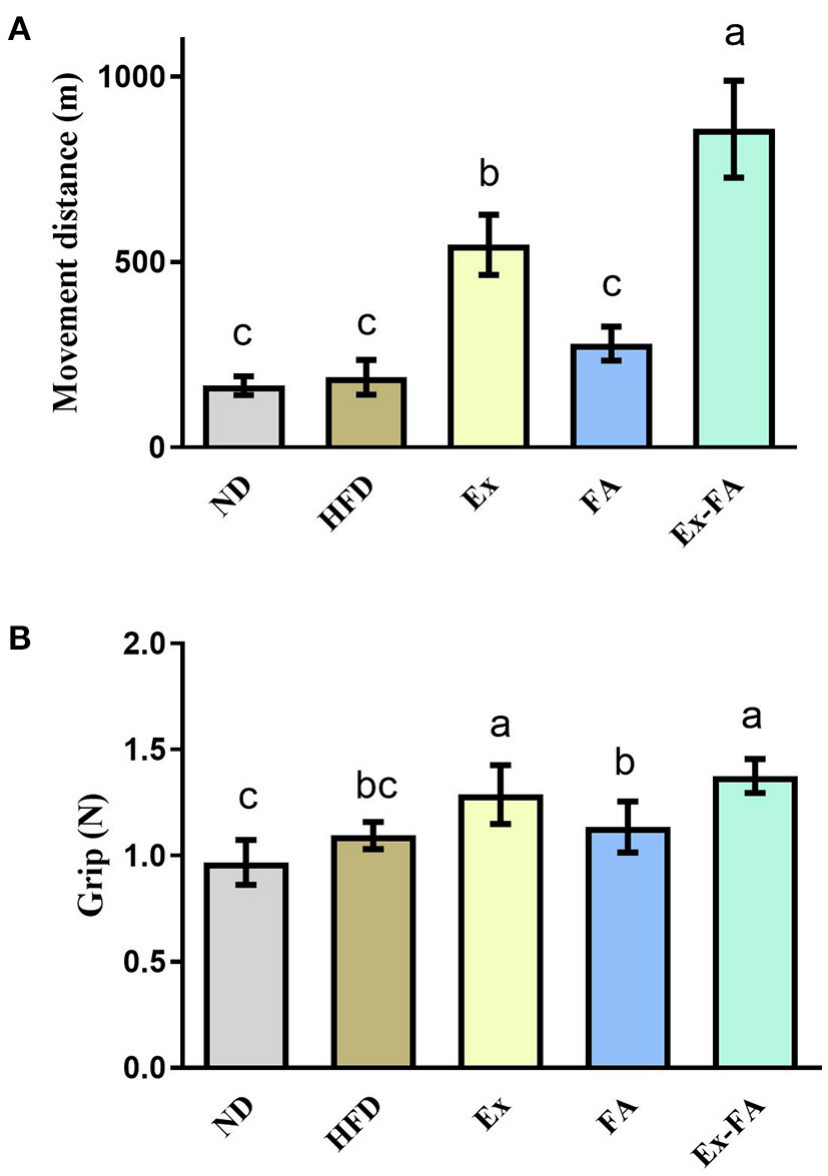
Effects of Ex, FA, and Ex-FA on exercise performance in HFD-fed mice. **(A)** Movement distance; **(B)** Grip strength. ND, normal diet group (control); HFD, high-fat diet group (model); Ex, high-fat diet with exercise; FA, high-fat diet with ferulic acid; Ex-FA, high-fat diet with exercise and ferulic acid. Data are expressed as mean ± SD (*n* = 4 for movement distance and *n* = 10 for grip strength). Means with different letters are significantly different (*P* < 0.05).

As shown in Figure 3A, when mice ran to exhaustion, the movement distance of mice in the Ex group was significantly longer than that of non-Ex mice in the ND, HFD, and FA groups (*P* < 0.05). FA administration alone exhibited no obvious effect on the movement distance compared to the ND and HFD group, but the movement distance of mice after FA consumption combined with Ex was significantly raised in comparison with that of mice in the Ex group by 57.1% (*P* < 0.05; Figure 3A). Besides, mice in the Ex and Ex-FA groups showed a markedly stronger grip force than mice in the other groups (*P* < 0.05; Figure 3B).

### Effects of Ex, FA, and Ex-FA on lipid levels in the serum, liver, and skeletal muscle of HFD-fed mice

In contrast to the ND group, the HFD supplementation led to a remarkable increase in the serum TG, TC, and LDL-C levels and a marked decrease in the serum HDL-C level (*P* < 0.05; Table 1). When the HFD-induced mice underwent regular Ex alone, the serum TG, TC, and LDL-C concentrations were significantly decreased by 42.9, 14.4, and 27.3%, respectively, and the serum HDL-C concentration was significantly elevated by 26.2% (vs. HFD, *P* < 0.05; Table 1). Besides, FA treatment alone exhibited notable effects in lowering the serum TG and TC levels compared to the HFD group (*P* < 0.05; Table 1). When the HFD-induced mice were administrated with both Ex and FA, the serum TG and LDL-C levels were significantly lower than those of mice in the HFD group by 34.7 and 22.7%, respectively, and the serum HDL-C level was significantly higher than that of mice in the HFD group by 29.5% (*P* < 0.05; Table 1).

**Table 1:** Effects of Ex, FA, and Ex-FA on lipid profiles in the serum, liver, and skeletal muscle in HFD-fed mice.

	**ND**	**HFD**	**Ex**	**FA**	**Ex-FA**
**Serum**					
TG (mmol/L)	0.34 ± 0.08^b^	0.49 ± 0.11^a^	0.28 ± 0.10^b^	0.33 ± 0.06^b^	0.32 ± 0.13^b^
TC (mmol/L)	3.41 ± 0.32^b^	4.10 ± 0.28^a^	3.51 ± 0.66^b^	3.46 ± 0.47^b^	3.72 ± 0.42^ab^
HDL-C (mmol/L)	2.87 ± 0.18^a^	2.37 ± 0.60^b^	2.99 ± 0.28^a^	2.72 ± 0.31^ab^	3.07 ± 0.36^a^
LDL-C (mmol/L)	0.30 ± 0.05^b^	0.44 ± 0.07^a^	0.32 ± 0.10^b^	0.38 ± 0.08^ab^	0.34 ± 0.06^b^
**Liver**					
TG (μmol/g pro)	31.40 ± 9.77^bc^	42.05 ± 7.75^a^	37.73 ± 7.28^ab^	32.16 ± 5.26^bc^	26.30 ± 6.58^c^
TC (μmol/g pro)	8.90 ± 1.09^a^	10.19 ± 1.92^a^	9.34 ± 1.34^a^	8.51 ± 0.63^a^	8.85 ± 2.49^a^
FFA (μmol/g pro)	22.86 ± 3.87^bc^	31.60 ± 8.01^a^	29.21 ± 7.15^ab^	20.28 ± 5.88^c^	21.30 ± 8.25^bc^
**Skeletal muscle**					
TG (μmol/g pro)	75.41 ± 4.05^bc^	83.34 ± 2.72^a^	80.35 ± 7.1^ab^	76.66 ± 3.46^abc^	72.26 ± 7.61^c^
TC (μmol/g pro)	2.73 ± 0.73^b^	5.40 ± 1.58^a^	4.81 ± 1.66^a^	2.98 ± 0.47^b^	3.11 ± 1.30^b^
FFA (μmol/g pro)	3.30 ± 1.25^b^	5.14 ± 1.19^a^	3.51 ± 1.06^b^	3.64 ± 0.89^b^	3.66 ± 0.78^b^

*ND, normal diet group (control); HFD, high-fat diet group (model); Ex, high-fat diet with exercise; FA, high-fat diet with ferulic acid; Ex-FA, high-fat diet with exercise and ferulic acid. Data are expressed as mean ± SD (n = 9 for skeletal muscle and n = 10 for serum and liver). Means with different letters are significantly different (P < 0.05)*.

In this study, the HFD consumption led to an obvious increment in the hepatic TG and FFA concentrations (vs. ND, *P* < 0.05; Table 1), which was remarkably attenuated by FA administration (*P* < 0.05; Table 1). In addition, the hepatic TG and FFA concentrations were not significantly influenced by Ex alone, but markedly decreased by the combined treatment of Ex and FA (vs. HFD, *P* < 0.05; Table 1).

Moreover, the contents of TG, TC, and FFA in the skeletal muscle of HFD-fed mice were significantly higher than those in the ND group (*P* < 0.05; Table 1). In contrast to the HFD group, Ex only remarkably lowered the muscular FFA level, and FA administration significantly decreased the muscular TC and FFA levels (*P* < 0.05; Table 1). However, after Ex is combined with FA administration, the HFD-induced lipid deposition in the skeletal muscle could be significantly inhibited (*P* < 0.05; Table 1). The muscular TG, TC, and FFA concentrations of mice in the Ex-FA group were significantly lower than those in the HFD group by 13.3, 42.4, and 28.8%, respectively (*P* < 0.05; Table 1).

### Effects of Ex, FA, and Ex-FA on oxidative stress status in HFD-fed mice

**Figure 4:**
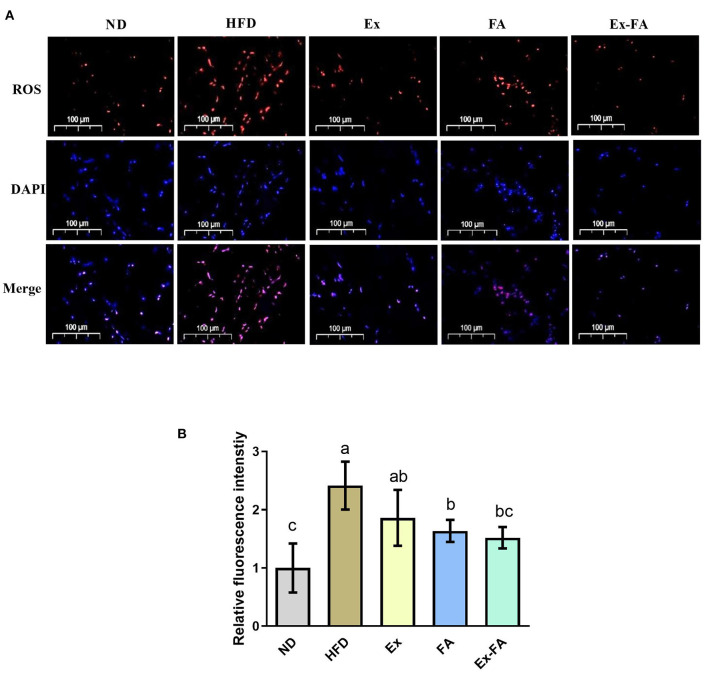
Effects of Ex, FA, and Ex-FA on the ROS level in HFD-fed mice. **(A)** ROS and DAPI staining of skeletal muscle sections (scale bar =100 μm). **(B)** Relative fluorescence intensity. Nucleus labeled with DAPI were blue and ROS positive areas were red. ND, normal diet group (control); HFD, high-fat diet group (model); Ex, high-fat diet with exercise; FA, high-fat diet with ferulic acid; Ex-FA, high-fat diet with exercise and ferulic acid. Data are expressed as mean ± SD (*n* = 6). Means with different letters are significantly different (*P* < 0.05).

The ROS level in the skeletal muscle under different treatments were observed by fluorescence staining (Figure 4A). Mice in the HFD group showed significantly higher ROS level compared to the ND group (*P* < 0.05; Figure 4B). The administrations of Ex, FA, and Ex-FA exhibited a similar tendency to lower the ROS level induced by HFD, while only FA and Ex-FA treatments markedly suppressed the ROS level (vs. HFD, *P* < 0.05; Figure 4B).

**Figure 5:**
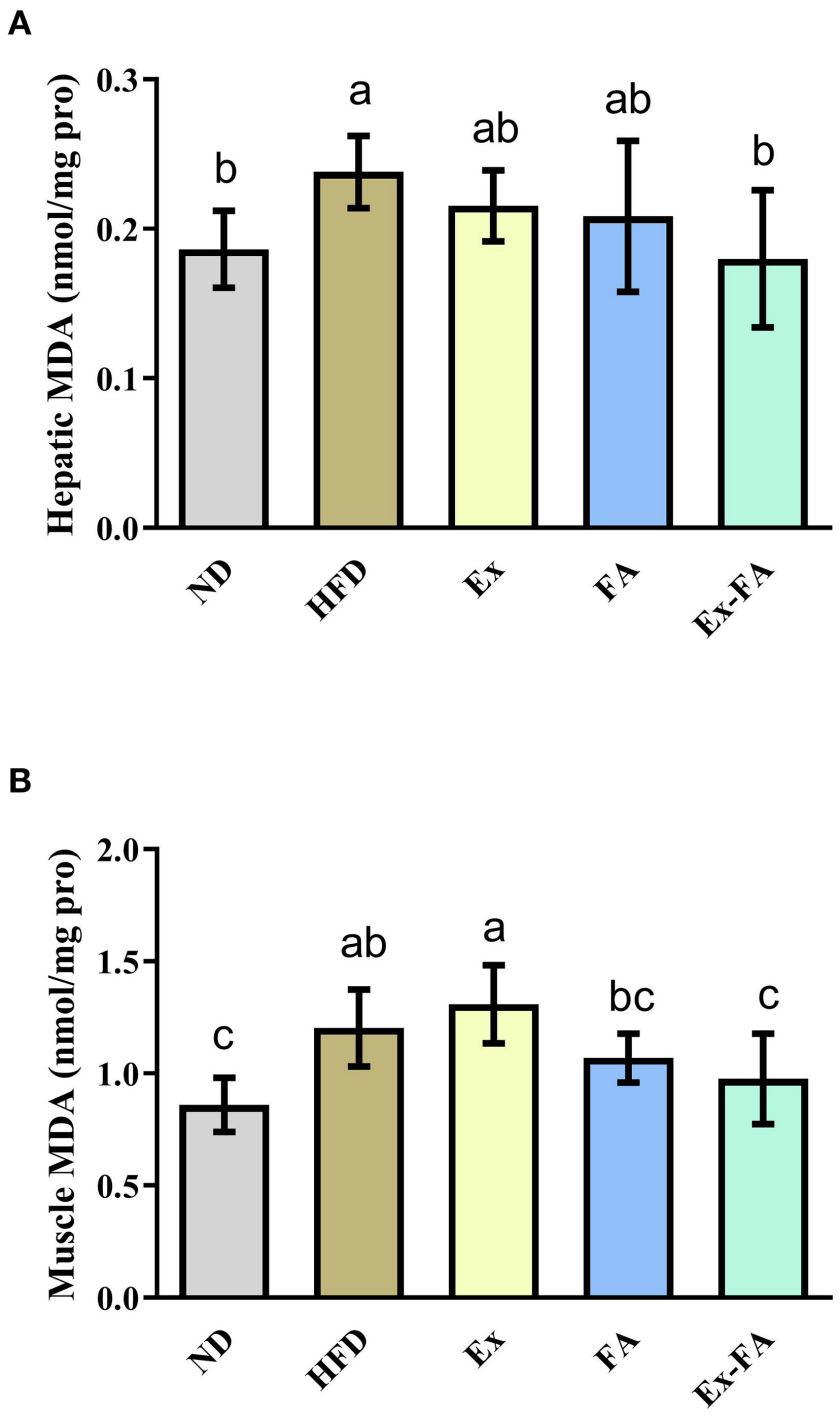
Effects of Ex, FA, and Ex-FA on the MDA level in the liver **(A)** and skeletal muscle **(B)** of HFD-fed mice. ND, normal diet group (control); HFD, high-fat diet group (model); Ex, high-fat diet with exercise; FA, high-fat diet with ferulic acid; Ex-FA, high-fat diet with exercise and ferulic acid. Data are expressed as mean ± SD (*n* = 10 for liver and *n* = 9 for muscle). Means with different letters are significantly different (*P* < 0.05).

Compared with mice in the ND group, mice in the HFD group showed significantly higher hepatic and muscular MDA levels (*P* < 0.05; Figure 5), indicative of lipid peroxidation. Mice induced by the combined treatment of Ex and FA possessed less hepatic and muscular MDA concentration than mice in the HFD group by 25.0 and 18.3%, respectively (*P* < 0.05; Figure 5).

### Multivariate analysis of metabolites

**Figure 6:**
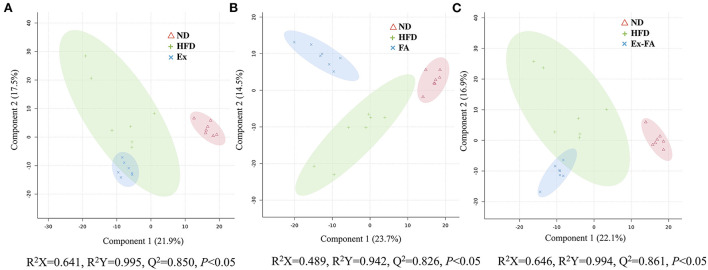
PLS-DA score plots. **(A)** The PLS-DA score plot of ND, HFD, and Ex groups; **(B)** The PLS-DA score plot of ND, HFD, and FA groups; **(C)** The PLS-DA score plot of ND, HFD, and Ex-FA groups. ND, normal diet group (control); HFD, high-fat diet group (model); Ex, high-fat diet with exercise; FA, high-fat diet with ferulic acid; Ex-FA, high-fat diet with exercise and ferulic acid.

**Figure 7:**
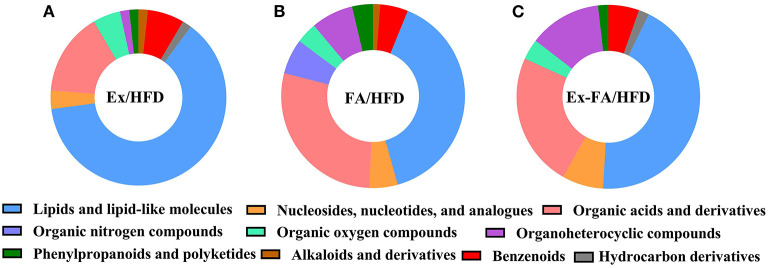
HMDB classification of the differential metabolites. **(A)** Ex vs. HFD; **(B)** FA vs. HFD; **(C)** Ex-FA vs. HFD. HFD, high-fat diet group (model); Ex, high-fat diet with exercise; FA, high-fat diet with ferulic acid; Ex-FA, high-fat diet with exercise and ferulic acid.

The results of PLS-DA are shown in Figure 6, and the R^2^X, R^2^Y, and Q^2^ values were shown to indicate the validness of the PLS-DA model. The differential metabolites between the HFD and other treatments were identified using the HMDB database and classified by the HMDB superclass. The metabolites of mice under different treatments showed diverse compositions. About 62.7% of the differential metabolites between the HFD and Ex groups were lipids and lipid-like molecules, followed by organic acids and derivatives accounting for about 15.3% (Figure 7A). However, for the differential metabolites between the HFD and FA groups, only 39.5% were lipids and lipid-like molecules, and about 28.4% were organic acids and derivatives (Figure 7B). Similarly, the proportion of lipids and lipids-like molecules in the differential metabolites between the HFD and Ex-FA groups was 43.6%, and the proportion of organic acids and derivatives of them was 23.6% (Figure 7C).

**Figure 8:**
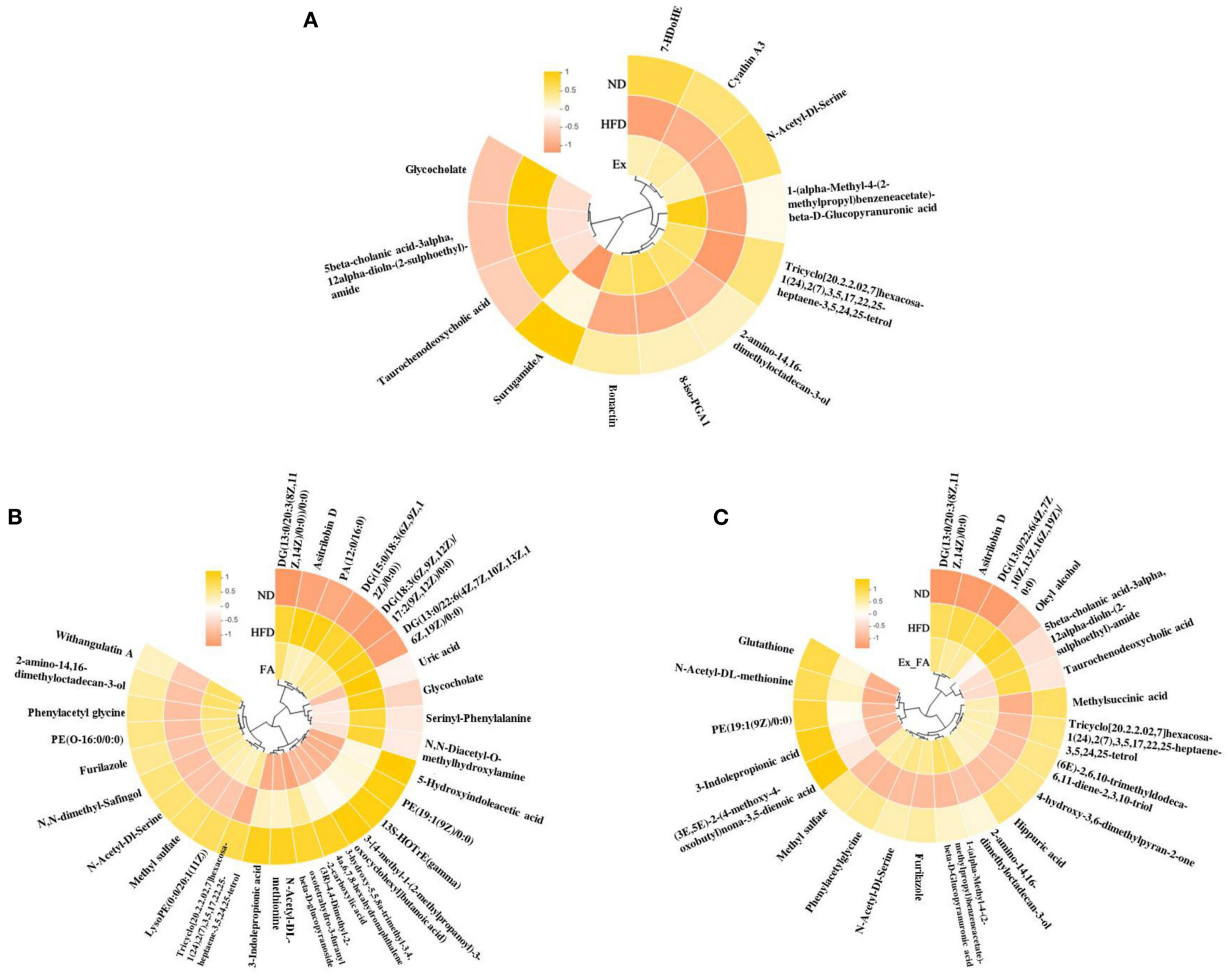
Heatmaps of the metabolites. **(A)** The common metabolites of ND, HFD, and Ex. **(B)** The common metabolites of ND, HFD, and FA. **(C)** The common metabolites of ND, HFD, and Ex-FA. ND, normal diet group (control); HFD, high-fat diet group (model); Ex, high-fat diet with exercise; FA, high-fat diet with ferulic acid; Ex-FA, high-fat diet with exercise and ferulic acid.

The heatmaps in Figure 8 showed the common metabolites in the ND, HFD, and treatment groups. In this study, a total of 189 differential metabolites were identified between the ND and HFD groups, and some of them were reversely changed by the treatments of Ex, FA, and Ex-FA, respectively (Supplementary Tables S1–S3). Between the two groups, the fold change of more than one was regarded as a significant increase in metabolites levels, while the fold change of less than one was regarded as a significant reduction in metabolites levels. Among the differential metabolites between the ND and HFD groups, there were 10 metabolites that were markedly varied by Ex administration, 19 markedly varied by FA supplementation, and 15 markedly varied by combined treatment of Ex and FA (Supplementary Tables S1–S3).

**Figure 9:**
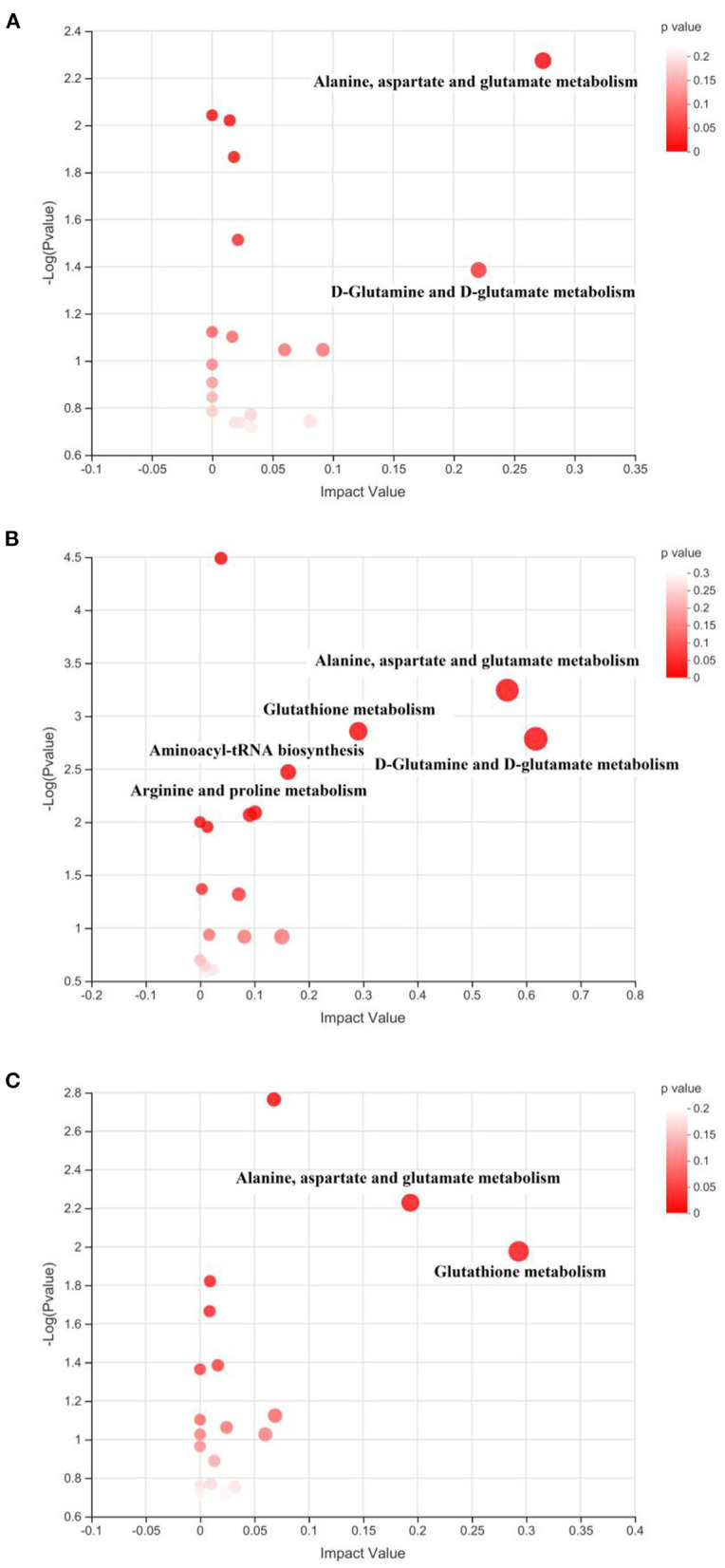
Topological analysis of the metabolic pathways. **(A)** Ex vs. HFD; **(B)** FA vs. HFD; **(C)** Ex-FA vs. HFD. HFD, high-fat diet group (model); Ex, high-fat diet with exercise; FA, high-fat diet with ferulic acid; Ex-FA, high-fat diet with exercise and ferulic acid.

The identified differential metabolites between the HFD group and different treatments were further applied for the pathway enrichment and topology analysis. As shown in Figure 9, each spot represents one potential metabolic pathway that is involved in the regulation of the differential metabolites. In this study, the metabolic pathway with a VIP value of more than 0.1 and a *P*-value of <0.05 were considered the important ones that significantly influenced the metabolism, as shown in Table 2. The result suggested that the key disturbed pathways were alanine, aspartate, and glutamate metabolism and D-Glutamine and D-glutamate metabolism between the HFD and Ex groups; D-Glutamine and D-glutamate metabolism, alanine, aspartate, and glutamate metabolism, glutathione metabolism, aminoacyl-tRNA biosynthesis, and arginine and proline metabolism between the HFD and FA groups; glutathione metabolism and alanine, aspartate and glutamate metabolism between the HFD and Ex-FA groups (Table 2).

**Table 2:** KEGG topological analysis.

	**Pathways**	**VIP value**	***P-*** **value**
**Ex/HFD**			
1	Alanine, aspartate and glutamate metabolism	0.27	0.01
2	D-glutamine and D-glutamate metabolism	0.22	0.04
**FA/HFD**			
1	D-glutamine and D-glutamate metabolism	0.62	<0.01
2	Alanine, aspartate and glutamate metabolism	0.57	<0.01
3	Glutathione metabolism	0.29	<0.01
4	Aminoacyl-tRNA biosynthesis	0.16	<0.01
5	Arginine and proline metabolism	0.10	<0.01
**Ex-FA/HFD**			
1	Glutathione metabolism	0.29	0.01
2	Alanine, aspartate and glutamate metabolism	0.19	0.01

*VIP value > 0.1 and P-value < 0.05 were listed. HFD, high-fat diet group (model); Ex, high-fat diet with exercise; FA, high-fat diet with ferulic acid; Ex-FA, high-fat diet with exercise and ferulic acid*.

## Discussion

Although Ex has been recognized as a healthy lifestyle, the Ex-induced oxidative stress cannot be ignored. FA is a well-known phenolic acid with antioxidant capacity and numerous health benefits. As a natural antioxidant, whether FA could maintain the redox homeostasis caused by Ex and have a synergistical effect with Ex on antiobesity remained unclear. Therefore, in the present study, the antiobesity effect of regular Ex, FA, and the combined treatment were compared in HFD-induced mice. The dosage of FA in this study was 136 mg/kg body weight in mice, which was approximately equal to a daily dosage of 660 mg per 60 kg adult (20). It has been estimated that a person's intake of polyphenols from a daily diet was about 1 g per day (21). Hence, the dosage of FA for the present research was within the reasonable range.

According to the present results, the administrations of Ex, FA, and Ex-FA exhibited similar effects in inhibiting the increase of the body weight and white fat tissue percentages induced by HFD (Figures 1, 2). However, they showed different effects in regulating the lipid levels in the serum, liver, and skeletal muscle. As shown in Table 1, regular Ex for 13 weeks significantly alleviated HFD-induced dyslipidemia but only markedly lowered the muscular FFA concentration. Nevertheless, FA treatment provoked a beneficial effect in reducing the serum TG and TC levels and a more comprehensive effect in decreasing the lipids levels in the liver and skeletal muscle than Ex. Therefore, Ex and FA may modulate the lipid metabolism and deposition through different ways. Hepatic lipid accumulation was the typical symptom of the non-alcohol fatty liver disease (NAFLD) (22), and Ex has been widely recommended to be an effective therapy for the management of NAFLD (23). However, the recent study indicated that although Ex could increase the energy utilization, the improvement of NAFLD was probably due to the changes in the features of hepatic lipid droplets rather than the decrease of the lipid contents (24). Therefore, Ex might achieve the hepatoprotective effect without the reduction of the hepatic lipid concentrations. Moreover, an isotopic tracking experiment showed that about 60% of the hepatic lipids came from the fatty acids released from adipocytes (25). It has been proved that FA could diminish differentiation and lipid accumulation in 3T3-L1 adipocytes (26) and regulate lipid metabolism in human adipocytes (27). Therefore, considering the adipocyte homeostasis induced by FA treatment, FA may show a potential inhibitory effect on hepatic lipid deposition. Besides, obesity was often accompanied by intramuscular lipid accumulation, further triggering insulin resistance and type 2 diabetes (28). In the present study, the TG, TC, and FFA levels in the skeletal muscle were significantly increased by HFD treatment, while muscular TC and FFA levels were markedly reduced by FA supplementation (Table 1). Similarly, a previous research also indicated that the inhibitory effect of FA on the HFD-induced muscular TG accumulation in obese mice was found and possibly achieved by elevating the protein expression levels of insulin receptor substrate-1, phosphatidylinositol 3-kinase, and phosphorylated-protein kinase B (17). Lifestyle interventions, including Ex, dietary, and behavior modification, may work together to achieve the hepatoprotective effect (29). Food rich in polyphenols, like green tea, combined with voluntary Ex could produce a more efficient effect on mitigating NAFLD than Ex alone (30). Consistent with this, the present study demonstrated that less hepatic TG concentration was found in the Ex-FA group compared to that in the Ex group (Table 1), which implied that the synergetic action of FA and Ex exerted a more outstanding benefit on liver protection.

Long-term HFD intake would produce excessive ROS, leading to systemic oxidative stress and lipid peroxidation, thus triggering oxidative damage (31, 32). In this study, the long-term HFD feeding significantly generated higher ROS level in the skeletal muscle and MDA concentration in the liver and skeletal muscle (Figures 4, 5), which indicated that HFD consumption caused oxidative stress. Ex showed a dual effect in the regulation of the body's antioxidant system, mainly depending on its duration and intensity. Briefly, moderate Ex could improve the redox homeostasis (33), while intensive and exhausted Ex could enhance lipid peroxidation and lead to oxidative damage (34). The previous study reported that the 8-week endurance training could suppress the obesity-induced oxidative stress in HFD-induced rats (35). Inconsistently, Ex under the specific intensity in this research showed an insignificant effect on the ROS scavenging and MDA reduction (Figures 4, 5). The differences in Ex duration, training program, and animal species may account for the discrepancy in these results. Besides, there was no clear definition of animal Ex intensity until now. As the ROS level in HFD-fed mice was not elevated after Ex alone, it could be speculated that the Ex intensity in this study was likely to moderate. Compared with Ex alone, treatment of Ex combined with FA markedly decreased the ROS level and MDA concentration in the liver and muscle (vs. HFD; Figures 4, 5). Among the chemicals with similar structures, FA showed the best antioxidant activity *in vitro* (36). Published research also indicated that FA treatment could alleviate HFD-induced oxidative stress, exhibiting the antioxidant capacity *in vivo* (37, 38). Therefore, FA administration could enhance the antioxidant system in exercised mice. Besides, body fatigue was closely related to oxidative stress (39), which might result in different Ex performances. FA administration could increase the activities of hepatic antioxidant enzymes and achieved a stimulatory effect with Ex to enhance endurance and reduce fatigue (40, 41). Consistent with this, mice in the Ex-FA group possessed significantly longer movement distance than mice in the other groups, indicating the antifatigue effect caused by the combined administration of Ex and FA (Figure 3).

Metabolites' differences could partially illustrate the change in the physiological activities *in vivo*. In this study, the HFD-fed mice after Ex, FA, and Ex-FA administrations showed different metabolic profiles. The findings in this study suggested that some of the differential metabolites might explain the mechanism of the anti-obesity effects under Ex, FA, and Ex-FA treatments.

Bile acid was closely associated with the catabolism of cholesterol, and contributed to the regulation of lipid metabolism (42). The circulating bile acid level was increased in obese people (43). In high-fat and high-cholesterol diet condition, the synthesis of bile acid was upregulated (44). Taurochenodeoxycholic acid (TCDCA) was a kind of conducted bile acid, which involved in the synthesis and secretion of bile acid, as well as cholesterol metabolism (45, 46). Glycocholate was another biomarker involved in the synthesis and excretion of bile acid. In this study, the abundance of TCDCA and glycocholate in serum were significantly increased by HFD ingestion while markedly reduced in the Ex group (Supplementary Table S1). It has been proved that Ex could decrease the serum bile acid concentration in obese people (43), which is probably achieved by increasing the excretion of it under exercise (47). Furthermore, the abundance of TCDCA and glycocholate in the serum also declined in the Ex-FA group and FA group, respectively (Supplementary Tables S2, S3), indicating the regulation of bile acid by FA intervention. In addition, the previous studies have found that the hydrophobic interactions between polyphenol compounds and bile acid could inhibit the solubility of micelle cholesterol (48) and absorption of bile acid into the colon (49), resulting in a higher bile acid concentration in feces (50).

Diacylglycerol (DG) was an endogenous intermediate in lipid metabolism and elevated in obese patients, further leading to insulin resistance (51, 52). In this study, several DG compounds were identified as the differential metabolites between the ND and HFD groups. As shown in Supplementary Table S2, HFD intake elicited an increase of the abundances of DG (13:0/20:3(8Z, 11Z, 14Z)/0:0), DG (13:0/22:6(4Z, 7Z, 10Z, 13Z, 16Z, 19Z)/0:0), DG (15:0/18:3(6Z, 9Z, 12Z)/0:0), and DG (18:3(6Z, 9Z, 12Z)/17:2(9Z, 12Z)/0:0), which were significantly reversed by FA administration. Ex-FA administration also exhibited a reducing effect on the abundance of DG (13:0/20:3(8Z, 11Z, 14Z)/0:0) (Supplementary Table S3). This result was in accordance with the previous research that the rice bran rich in FA could decrease the DG compounds in high-energy-induced rats (53). Therefore, the antiobesity effect of FA might be partially attributed to the regulation of intermediates in lipid metabolism.

Additionally, hyperuricemia is one of the obesity-related metabolic disorders, and xanthine oxidase is recognized as the target against hyperuricemia (54). FA has been proved to be a xanthine oxidase inhibitor (55). Therefore, the abundance of serum uric acid was significantly elevated by HFD but notably decreased by FA treatment (Supplementary Table S2).

**Figure 10:**
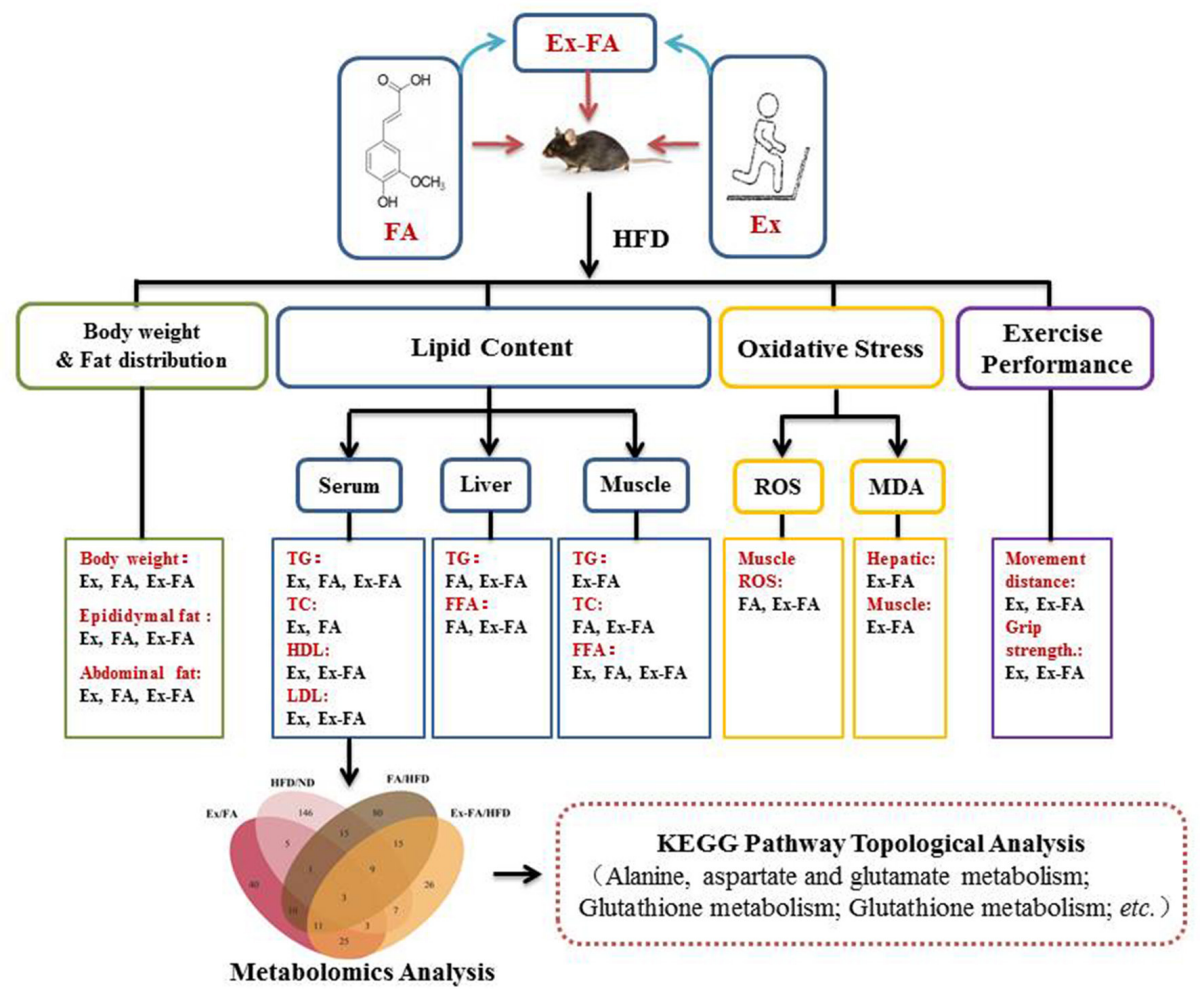
A brief schematic of the regulatory effect of Ex, FA, and Ex-FA in HFD-induced mice. HFD, high-fat diet group (model); Ex, high-fat diet with exercise; FA, high-fat diet with ferulic acid; Ex-FA, high-fat diet with exercise and ferulic acid.

The topology analysis of metabolic pathways was carried out to further evaluate the possible mechanisms of the antiobesity effect of Ex, FA, and Ex-FA treatments. As shown in Table 2 and Figure 9, alanine, aspartate and glutamate metabolisms were identified as the common pathways in all the treatment groups, and several different pathways were analyzed to be specific in different groups. Although pathways directly related to lipid metabolism were not the dominant ones in this topology analysis, the biomarkers of lipids metabolism were found to be significantly different in FA or Ex-FA group in comparison with the HFD group. This is probably because the serum metabolic profile reflects the systemic metabolic situation. The major organs of lipid metabolism, such as the liver and adipose tissue, might be more appropriate for analyzing the metabolic profiles of lipid metabolism, which will be conducted in future studies. Based on all the above discussion, a brief schematic summarized the regulatory effect of Ex, FA, and Ex-FA in HFD-induced mice (Figure 10).

## Conclusion

In summary, this study demonstrated that Ex, FA, and Ex-FA showed different antiobesity effects in HFD-fed mice. They had a similar effect on body weight management. Ex could alleviate HFD-induced dyslipidemia, while FA could mitigate lipid deposition in the liver and muscle. Moreover, Ex-FA not only showed comprehensive effects on the regulation of the lipid contents in serum, liver, and skeletal muscle, but also exhibited enhancement effects on antioxidant ability and Ex capacity. Mice administered Ex, FA, and Ex-FA produced different metabolic profiles, which might be achieved through different metabolic pathways.

## Data availability statement

The original contributions presented in the study are included in the article/Supplementary material, further inquiries can be directed to the corresponding author.

## Ethics statement

This animal experimental protocol was approved by the Laboratory Animal Welfare and Ethics Committee of National Institute for Nutrition and Health, Chinese Center for Disease Control and Prevention.

## Author contributions

OW: project administration, data analysis, visualization, and writing. NZ: project administration and writing. CH: data collection. JH: supervision and manuscript revision. All authors contributed to the article and approved the submitted version.

## Funding

This study was funded by the Beijing Natural Science Foundation (6214047).

## Conflict of interest

The authors declare that the research was conducted in the absence of any commercial or financial relationships that could be construed as a potential conflict of interest.

## Publisher's note

All claims expressed in this article are solely those of the authors and do not necessarily represent those of their affiliated organizations, or those of the publisher, the editors and the reviewers. Any product that may be evaluated in this article, or claim that may be made by its manufacturer, is not guaranteed or endorsed by the publisher.
