# Supplementary material for: Regular exercise combined with ferulic acid exhibits antiobesity effect and regulates metabolic profiles in high-fat diet-induced mice
Source: Front Nutr. 2022 Jul 27;9:957321. doi: 10.3389/fnut.2022.957321 (PMC9363793; doi:10.3389/fnut.2022.957321)
Supplement: Supplementary file 1 [file Data_Sheet_1.docx]

Supplementary Material

**Table S1.** The significantly corrected serum metabolites in HFD-fed mice treated with exercise.

| No. | Metabolites | Formula | Mode | m/z | Fold change | | *P* value | |
| --- | --- | --- | --- | --- | --- | --- | --- | --- |
|  |  |  |  |  | HFD/ND | Ex/HFD | HFD/ND | Ex/HFD |
| 1 | Bonactin | C_21_H_36_O_7_ | - | 421.2214 | 0.96 | 1.05 | 0.04 | 0.02 |
| 2 | N-Acetyl-Dl-Serine | C_5_H_9_NO_4_ | - | 192.0511 | 0.89 | 1.09 | 0.02 | 0.04 |
| 3 | Glycocholate | C_26_H_43_NO_6_ | - | 464.3017 | 1.34 | 0.79 | <0.01 | 0.01 |
| 4 | 8-iso-PGA1 | C_20_H_32_O_4_ | + | 381.2000 | 0.96 | 1.05 | 0.05 | 0.01 |
| 5 | 5beta-cholanic acid-3alpha, 12alpha-dioln-(2-sulphoethyl)-amide | C_26_H_45_NO_6_S | + | 500.3020 | 1.17 | 0.88 | 0.01 | 0.02 |
| 6 | Tricyclo[20.2.2.02,7]hexacosa-1(24),2(7),3,5,17,22,25-heptaene-3,5,24,25-tetrol | C_26_H_34_O_4_ | + | 455.2129 | 0.96 | 1.04 | <0.01 | <0.01 |
| 7 | 2-amino-14,16-dimethyloctadecan-3-ol | C_20_H_43_NO | + | 314.3406 | 0.95 | 1.08 | 0.03 | 0.04 |
| 8 | Cyathin A3 | C_20_H_30_O_3_ | + | 319.2257 | 0.96 | 1.03 | 0.02 | 0.03 |
| 9 | Taurochenodeoxycholic acid | C_26_H_45_NO_6_S | + | 464.2815 | 1.11 | 0.92 | 0.03 | 0.04 |
| 10 | 1-(alpha-Methyl-4-(2-methylpropyl)benzeneacetate)-beta-D-Glucopyranuronic acid | C_19_H_26_O_8_ | - | 403.1402 | 0.84 | 1.34 | 0.03 | <0.01 |

ND: normal diet group (control); HFD: high-fat diet group (model); Ex: high-fat diet with exercise.

**Table S2.** The significantly corrected serum metabolites in HFD-fed mice treated with ferulic acid.

| No. | Metabolites | Formula | Mode | m/z | Fold change | | *P* value | |
| --- | --- | --- | --- | --- | --- | --- | --- | --- |
|  |  |  |  |  | HFD/ND | FA/HFD | HFD/ND | FA/HFD |
| 1 | Furilazole | C_11_H_13_C_l2_NO_3_ | - | 332.0247 | 0.90 | 1.11 | 0.03 | 0.04 |
| 2 | 2-amino-14,16-dimethyloctadecan-3-ol | C_20_H_43_NO | + | 314.3406 | 0.95 | 1.07 | 0.03 | 0.02 |
| 3 | Asitrilobin D | C_37_H_68_O_7_ | + | 647.4845 | 1.20 | 0.94 | <0.01 | 0.01 |
| 4 | DG(13:0/20:3(8Z,11Z,14Z)/0:0) | C_36_H_64_O_5_ | + | 577.4790 | 1.48 | 0.96 | <0.01 | 0.01 |
| 5 | DG(13:0/22:6(4Z,7Z,10Z,13Z,16Z,19Z)/0:0) | C_38_H_62_O_5_ | + | 599.4625 | 1.12 | 0.97 | <0.01 | 0.01 |
| 6 | DG(15:0/18:3(6Z,9Z,12Z)/0:0) | C_36_H_64_O_5_ | + | 621.4448 | 1.16 | 0.97 | <0.01 | 0.03 |
| 7 | DG(18:3(6Z,9Z,12Z)/17:2(9Z,12Z)/0:0) | C_38_H_64_O_5_ | + | 601.4790 | 1.14 | 0.97 | <0.01 | <0.01 |
| 8 | Glycocholate | C_26_H_43_NO_6_ | - | 464.3017 | 1.34 | 0.79 | <0.01 | 0.01 |
| 9 | LysoPE(0:0/20:1(11Z)) | C_25_H_50_NO_7_P | + | 490.3249 | 0.94 | 1.04 | 0.02 | 0.02 |
| 10 | Methyl sulfate | CH_4_O_4_S | - | 110.9750 | 0.88 | 1.10 | 0.01 | 0.03 |
| 11 | N,N-dimethyl-Safingol | C_20_H_43_NO_2_ | + | 330.3355 | 0.95 | 1.05 | 0.02 | 0.02 |
| 12 | N-Acetyl-Dl-Serine | C_5_H_9_NO_4_ | - | 192.0511 | 0.89 | 1.10 | 0.02 | 0.03 |
| 13 | PA(12:0/16:0) | C_31_H_61_O_8_P | + | 593.4136 | 1.22 | 0.93 | <0.01 | 0.01 |
| 14 | PE(O-16:0/0:0) | C_21_H_46_NO_6_P | + | 440.3125 | 0.96 | 1.05 | 0.02 | 0.01 |
| 15 | Phenylacetyl glycine | C_10_H_11_NO_3_ | + | 194.0807 | 0.94 | 1.06 | 0.01 | 0.02 |
| 16 | Serinyl-Phenylalanine | C_12_H_16_N_2_O_4_ | + | 253.1175 | 1.25 | 0.80 | 0.02 | 0.04 |
| 17 | Tricyclo[20.2.2.02,7]hexacosa-1(24),2(7),3,5,17,22,25-heptaene-3,5,24,25-tetrol | C_26_H_34_O_4_ | + | 455.2129 | 0.96 | 1.03 | <0.01 | <0.01 |
| 18 | Uric acid | C_5_H_4_N_4_O_3_ | + | 169.0351 | 1.02 | 0.97 | <0.01 | <0.01 |
| 19 | Withangulatin A | C_30_H_38_O_8_ | + | 540.2706 | 0.96 | 1.07 | 0.04 | 0.01 |

ND: normal diet group (control); HFD: high-fat diet group (model); FA: high-fat diet with ferulic acid.

**Table S3.** The significantly corrected serum metabolites in HFD-fed mice treated with exercise and ferulic acid.

| No. | Metabolites | Formula | Mode | m/z | Fold change | | P value | |
| --- | --- | --- | --- | --- | --- | --- | --- | --- |
|  |  |  |  |  | HFD/ND | Ex-FA/HFD | HFD/ND | Ex-FA/HFD |
| 1 | Furilazole | C_11_H_13_C_l2_NO_3_ | - | 322.0247 | 0.90 | 1.12 | 0.03 | 0.02 |
| 2 | (6E)-2,6,10-trimethyldodeca-6,11-diene-2,3,10-triol | C_15_H_28_O_3_ | - | 301.2025 | 0.93 | 1.07 | 0.02 | 0.04 |
| 3 | Oleyl alcohol | C_18_H_36_O | - | 313.2751 | 1.08 | 0.96 | <0.01 | 0.01 |
| 4 | 1-(alpha-Methyl-4-(2-methylpropyl)benzeneacetate)-beta-D-Glucopyranuronic acid | C_19_H_26_O_8_ | - | 403.1402 | 0.84 | 1.26 | 0.03 | <0.01 |
| 5 | Hippuric acid | C_9_H_9_NO_3_ | - | 178.0505 | 0.89 | 1.09 | 0.01 | 0.05 |
| 6 | N-Acetyl-Dl-Serine | C_5_H_9_NO_4_ | - | 192.0511 | 0.89 | 1.14 | 0.02 | 0.01 |
| 7 | 4-hydroxy-3,6-dimethylpyran-2-one | C_7_H_8_O_3_ | + | 105.0336 | 0.88 | 1.10 | 0.02 | 0.05 |
| 8 | 5beta-cholanic acid-3alpha, 12alpha-dioln-(2-sulphoethyl)-amide | C_26_H_45_NO_6_S | + | 500.302 | 1.17 | 0.85 | 0.01 | 0.02 |
| 9 | Asitrilobin D | C_37_H_68_O_7_ | + | 647.4845 | 1.20 | 0.97 | <0.01 | 0.02 |
| 10 | DG(13:0/20:3(8Z,11Z,14Z)/0:0) | C_36_H_64_O_5_ | + | 577.4790 | 1.48 | 0.97 | <0.01 | 0.03 |
| 11 | Tricyclo[20.2.2.02,7]hexacosa-1(24),2(7),3,5,17,22,25-heptaene-3,5,24,25-tetrol | C_26_H_34_O_4_ | + | 455.2129 | 0.96 | 1.03 | <0.01 | 0.02 |
| 12 | 2-amino-14,16-dimethyloctadecan-3-ol | C_20_H_43_NO | + | 314.3406 | 0.95 | 1.09 | 0.03 | 0.02 |
| 13 | Taurochenodeoxycholic acid | C_26_H_45_NO_6_S | + | 464.2815 | 1.11 | 0.89 | 0.03 | 0.02 |
| 14 | Phenylacetylglycine | C_10_H_11_NO_3_ | + | 194.0807 | 0.94 | 1.06 | 0.01 | 0.01 |
| 15 | Methyl sulfate | CH_4_O_4_S | - | 110.9750 | 0.88 | 1.12 | 0.01 | 0.01 |

ND: normal diet group (control); HFD: high-fat diet group (model); Ex-FA: high-fat diet with exercise and ferulic acid.
